# GluN2A-selective positive allosteric modulator-nalmefene-flumazenil reverses ketamine-fentanyl-dexmedetomidine-induced anesthesia and analgesia in rats

**DOI:** 10.1038/s41598-020-62192-8

**Published:** 2020-03-24

**Authors:** Chunzhu Li, Jia Yan, Dewei Tang, Jidong Zhu, Chen Huang, Yu Sun, Rong Hu, Hao Wang, Chaoying Fu, Yelin Chen, Hong Jiang

**Affiliations:** 1 Department of Anesthesiology, Shanghai Ninth People’s Hospital, Shanghai Jiao Tong University School of Medicine, Center for Specialty Strategy Research of Shanghai Jiao Tong University China Hospital Development Institute, Shanghai, 200011 China; 2 Interdisciplinary Research Center on Biology and Chemistry, Shanghai Institute of Organic Chemistry, Chinese Academy of Sciences, Shanghai, 201210 China; 3 University of Chinese Academy of Sciences, Beijing, 100049 China; 4 Center for Molecular Imaging, Shanghai University of Medicine & Health Sciences, Shanghai, China; 5 Department of Nuclear Medicine, Renji Hospital, School of Medicine, Shanghai Jiao Tong University, 160 Pujian Road, Pudong New District, Shanghai, 200127 China; 6 College of Medical Imaging and Shanghai Key Laboratory of Molecular Imaging, Shanghai University of Medicine and Health Sciences, Shanghai, 201318 China

**Keywords:** Disability, Pharmacodynamics, Preclinical research

## Abstract

Anesthetics are used to produce hypnosis and analgesic effects during surgery, but anesthesia for a long time after the operation is not conducive to the recovery of animals or patients. Therefore, finding appropriate treatments to counter the effects of anesthetics could enhance postoperative recovery. In the current study, we discovered the novel role of a GluN2A-selective positive allosteric modulator (PAM) in ketamine-induced anesthesia and investigated the effects of the PAM combined with nalmefene and flumazenil (PNF) in reversing the actions of an anesthetic combination (ketamine-fentanyl-dexmedetomidine, KFD). PAM treatment dose-dependently decreased the duration of the ketamine-induced loss of righting reflex (LORR). Compared with those in the KFD group, the duration of LORR and the analgesic effect of the KFD + PNF group were obviously decreased. Meanwhile, successive administration of PNF and KFD had no adverse effects on the cardiovascular and respiratory systems. Both the KFD group and the KFD + PNF group showed no changes in hepatic and renal function or cognitive function in rats. Moreover, the recovery of motor coordination of the KFD + PNF group was faster than that of the KFD group. In summary, our results suggest the potential application of the PNF combination as an antagonistic treatment strategy for anesthesia.

## Introduction

Ketamine, a noncompetitive NMDA receptor antagonist, is a dissociative general anesthetic^1,2^. Fentanyl is a synthetic µ-opioid receptor agonist used as a narcotic analgesic agent in general and regional anesthesia, since it exhibits a rapid onset, a short duration, and potent analgesic effects^3,4^. Dexmedetomidine, a selective α2-adrenoceptor agonist with profound sedative, analgesic, amnestic and anesthetic-sparing properties, is commonly used as an anesthetic adjuvant clinically^5,6^. In a previous study, we showed that the low-dose combination of ketamine, fentanyl and dexmedetomidine (KFD) offered safer and more efficient anesthesia than ketamine alone^7^. However, compared with ketamine, the KFD combination induced a longer duration of the loss of righting reflex (LORR)^7^. Hence, identification of appropriate treatments to reverse the effects of anesthetics could help reduce the risk caused by prolonged unnecessary anesthesia.

GNE-5729^8^, which we used in the present study, is a pyridopyrimidinone-based GluN2A subunit-selective positive allosteric modulator (PAM) of the NMDA receptor. Subunit-selective PAMs are new classes of NMDA receptor agents that act at several newly identified binding sites on the NMDA receptor complex, and they do not directly activate the NMDA receptor but enhance the NMDA receptor response to its agonists^9,10^. Moreover, compared with previously available agents, PAMs provide greater pharmacological control over NMDA receptor activity^11,12^. PAMs are less likely to be linked with off-target and other unwanted side effects, such as excitotoxicity, than other agents because of their enhanced subunit selectivity and lack of intrinsic efficacy in the absence of endogenous glutamate, glycine, or D-serine^11^. In contrast, an NMDA receptor agonist activates both proper and improper receptors and thus increases system noise and probably causes excitotoxicity^13,14^.

Nalmefene (NMF) is an opioid antagonist that is similar in structure and activity to the μ-opioid receptor antagonist naltrexone^15^. Nalmefene has many advantages relative to naltrexone, such as greater oral bioavailability, higher affinity for the μ-opioid receptor, and a longer half-life^16,17^. Nalmefene is used to completely or partially reverse the effects of natural and synthetic opioids^15,18^. Dougherty *et al*. found that nalmefene at 0.5 μg/kg could reverse analgesia in approximately 20% of patients receiving epidural fentanyl in dilute bupivacaine for postoperative pain control^19^. It has also been reported that nalmefene could more effectively reverse the LORR induced by carfentanil, a derivative of fentanyl, in rats than naloxone^20^. Flumazenil (FMZ) is a drug that acts at the benzodiazepine binding site on the GABA_A_ receptor. As ketamine-induced hypnosis in mice was shown to involve the GABA_A_ receptor^21,22^, and flumazenil could counteract the impairing effects of ketamine on recognition memory in rats^23^, we chose flumazenil as a candidate for the antagonistic formulations of KFD.

In the present study, using rat models of anesthesia and analgesia, we discovered that a PAM (GNE-5729) substantially antagonized the effects of ketamine. Moreover, the combination of the PAM, nalmefene, and flumazenil could significantly reverse sedation and analgesia in rats receiving KFD. To further elucidate the pharmacological effects of PNF, we also focused on the safety and effectiveness of PNF and found that successive treatment with KFD and PNF in rats had no effects on cardiorespiratory, hepatic, renal, and cognitive function.

## Materials and Methods

### Animals

Sprague-Dawley rats (5–6 weeks old, male or female) were used in this study. The animal experiments were approved by the Animal Care Committee of the Ninth People’s Hospital, Shanghai Jiao Tong University School of Medicine (Shanghai, China), and all methods were performed in accordance with the relevant guidelines. Rats were maintained in a temperature-controlled environment under a 12 h light/dark cycle (07:00 a.m. to 07:00 p.m.) and provided with food and water ad libitum.

### Anesthesia

Ketamine (Gutian Medical, Inc., Fujian, China; 50 mg/mL), fentanyl (Humanwell Pharmaceutical, Yichang, China; 0.05 mg/mL), dexmedetomidine (Guorui Medical, Inc., Sichuan, China; 0.1 mg/mL), nalmefene (Chengdu Tiantaishan Pharmaceutical Co., Ltd., Chengdu, China; 0.1 mg/mL), and flumazenil (Hainan Lionco Pharmaceutical Co., Ltd., Hainan, China; 0.1 mg/mL) were dissolved in a sterile saline solution (Chimin Pharmaceutical Co., Ltd., Zhejiang, China) before intraperitoneal injection (i.p.) at 10 mL/kg body weight. GNE-5729 (99.9%) is a potent positive allosteric modulator (PAM) of GluN2A-containing NMDA receptors. Due to its low solubility, this molecule was prepared in a corn oil solution and used for intragastric administration (i.g.) at 10 mL/kg. The drug doses were selected on the basis of previous studies with slight modifications^7,18,23^. All rats consistently responded to the drugs in the same way, and we did not observe any mortality throughout the study.

**Figure 1:**
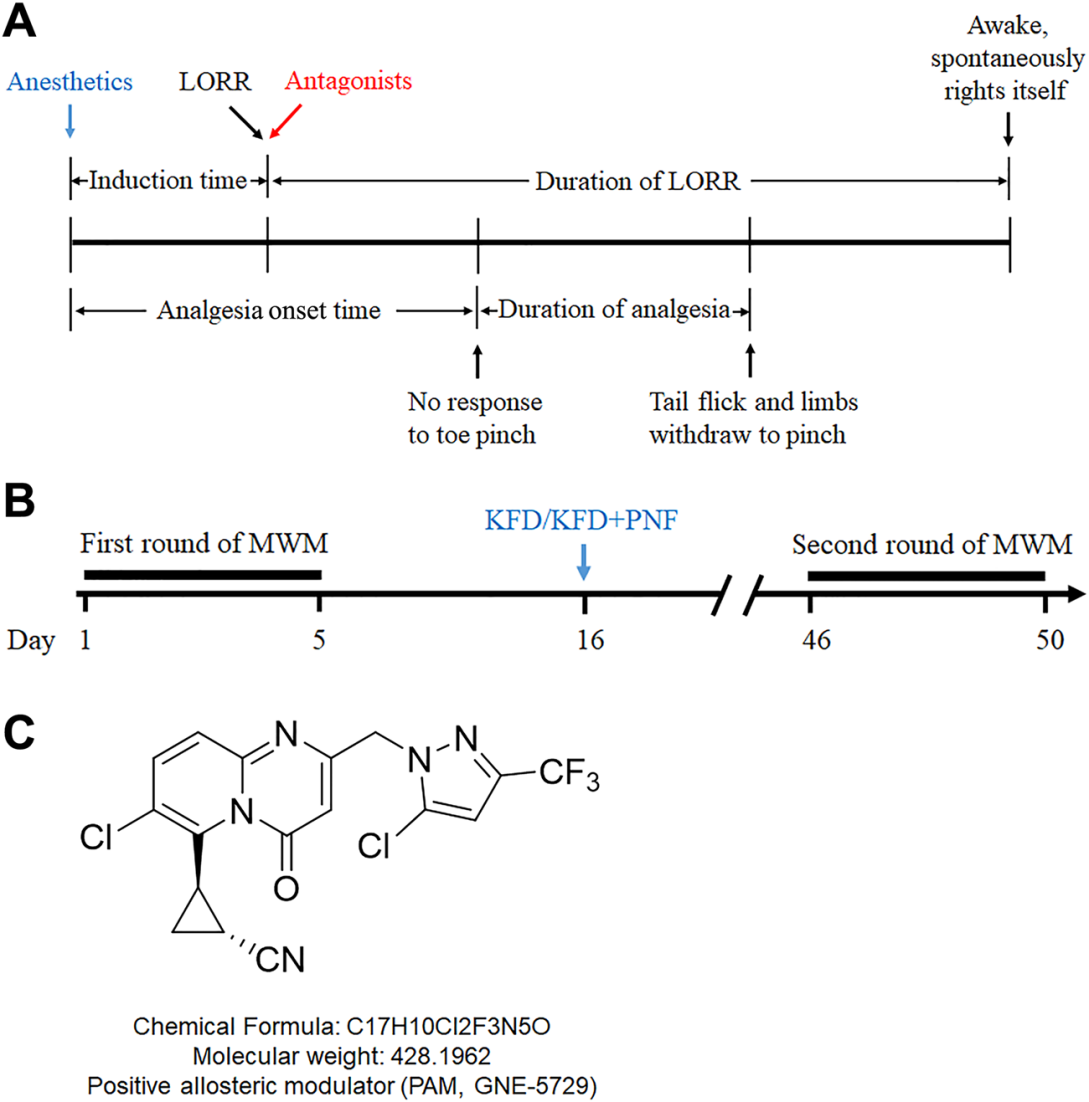
Schematic diagram of the evaluation of anesthesia and the MWM experimental design. (**A**) The anesthesia time was divided into four intervals. After the rats were anesthetized, antagonists were administered once the righting reflex was lost, and the durations of LORR and analgesia were recorded. (**B**) Experimental design of the MWM. KFD: combination of 10 mg/kg ketamine, 0.01 mg/kg fentanyl, and 0.1 mg/kg dexmedetomidine; the combination of anesthetics was administered in a single injection. PNF: combination of 2 mg/kg PAM, 0.1 mg/kg nalmefene, and 0.4 mg/kg flumazenil; the PAM was administered i.g. followed by injection of the nalmefene and flumazenil combination. (**C**) The structure of the PAM (GNE-5729).

The anesthetic and analgesic effects were evaluated by the duration of LORR and the absence of the pain reflex. After the rats were anesthetized, antagonists were administered once the righting reflex was lost, and the duration of LORR and analgesia were recorded. LORR was defined as having occurred when the rat did not right itself for at least 10 s after being placed in the supine position. Then, a toe pinch test was performed. A warming blanket was used to maintain the animal’s body temperature at approximately 37 °C. As shown in Fig. 1A, the anesthesia time was divided into the following intervals: 1. the induction time defined as the time from the anesthetic(s) administration to complete LORR; 2. the analgesia onset time defined as the time from the anesthetic(s) administration to complete no response to toe pinch; 3. the duration of analgesia time defined as the time from the absence of the limb withdrawal reflex to the return of the tail flick and limb withdrawal reflex; and 4. the duration of LORR. Recovery from the LORR was defined as having occurred when the rat spontaneously righted itself ^24^.

### Surface electrocardiography (ECG)

Once anesthetic administration was completed and the righting reflex was lost, the rats were placed in the supine position on a Mouse Monitor S (Indus Instruments) heating pad with needle ECG leads and recorded according to the manufacturer’s recommendations. The respiratory rate and SpO_2_ were measured using the same equipment.

### Serological analysis

Rats were anesthetized with isoflurane, and blood was collected from the fundus vein of each animal before saline or KFD administration (0 h) and at 0.5, 12, 24, and 48 h after saline or KFD injection. Then, the blood samples were centrifuged at 4 °C and 4000 g for 10 min to obtain 200 μL of serum for testing. Serum alanine aminotransferase (ALT), aspartate transaminase (AST), urea, and creatinine (CREA) were determined using an automatic Hitachi Clinical Analyzer Model 7080 (Hitachi High-Technologies Corporation, Tokyo, Japan).

### Morris water maze

The Morris water maze (MWM) apparatus included a black circular pool with the following dimensions: 160 cm in diameter and 50 cm in height and filled with warm water at 23 °C. A 10-cm-diameter platform was submerged 2 cm below the water surface. The acquisition phase included three trials each day for four consecutive days. During each trial, the rats were released into the water from a specific starting point located at the quadrant opposite the platform. The time to find the platform and the distance swum before reaching the platform were recorded, and the rats were allowed to swim freely until they reached the platform in 2 min and stayed on it for 30 s. If the rats did not locate the platform, they were gently guided to the platform and were allowed to rest on it for 30 s, and the latency was recorded as 120 s. On the 5th day, the rats were tested on a spatial probe trial in which the platform was removed, and they were allowed to swim freely for 120 s. The time to reach the platform initially, the ratio of time spent in the range around the platform as determined by software, and the number of times that the rats crossed the platform were recorded.

For determination of whether the spatial learning and memory of animals were at similar levels and for elimination of non-drug effects, rats (5 weeks, n = 8–11) underwent the MWM test (Days 1–5) before administration. We choose younger animals because developing brains are more vulnerable to anesthetics^25,26^, and GluN2A (target of PAM) is expressed starting two weeks after birth^27,28^; therefore, we chose the earliest possible age to assess the safety of the drugs. Training in the MWM might induce stress in the animals, which could interfere with the drug effects; therefore, we waited ten days for drug administration. Then, the rats were administered saline or drugs (Day 16). For KFD + PNF treatment, the rats received PNF administration once they lost the righting reflex after KFD injection. Because the long-term side effects of anesthetics usually emerge after a few weeks^29–31^, we reassessed the animals 30 days after the drug treatment (Days 46–50) (Fig. 1B).

### Rotarod test

Motor coordination and balance were assessed by the rotarod test. Before the experiment began, all rats were trained to walk on the automated rotarod device (Sans biotech Co., Ltd., Jiangsu, China) to achieve stable performance. The speed for the training sessions was set at 25 rpm. On the first three days (training days), the animals were placed on the rotarod until they could stay on the device for 2 min, whereby they were quickly placed back on the rod when contact with the rotating drum was not maintained. On the fourth day (testing day), the rats were administered KFD or a combination of KFD and PNF. The duration from the rat spontaneously righting itself to walking for 30 cm was recorded, and then, the rat was placed on the rod. The time when the rat spontaneously righted itself to remain on the rotating rod (25 rpm) for 20 s was measured.

### Statistical analysis

Data are presented as the mean ± SD (standard deviation). Student’s *t*-test (two-tailed) or one-way analysis of variance was employed to analyze data unless otherwise mentioned, and Tukey’s test was used for corrections for multiple comparisons. All statistical analyses were performed using SPSS v.11.5 software (SPSS, Chicago, IL, USA). A *P*-value of less than 0.05 was considered statistically significant.

## Results

### The PAM substantially reduced the ketamine-induced duration of LORR

**Figure 2:**
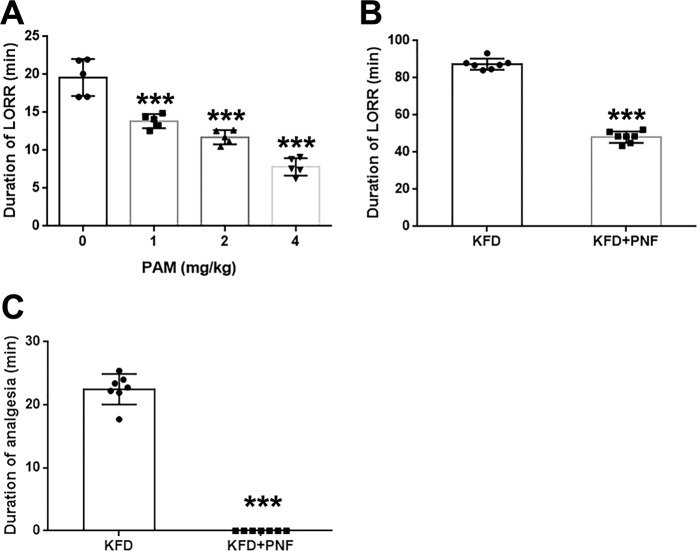
The reversal effects of antagonists on the functions of anesthetics. After rats were anesthetized, antagonists or saline were administered once the righting reflex was lost, and the durations of LORR and analgesia were recorded. (**A**) The PAM dose-dependently decreased the duration of LORR induced by ketamine. Ketamine: 50 mg/kg. Significant differences were determined by one-way analysis of variance, mean ± SD, n = 5, male. ***P < 0.001 vs. the ketamine group. (**B**) PNF reduced the KFD-induced duration of LORR (*P* = 1.56E-11). (**C**) PNF completely reversed the analgesic effect of KFD (*P* = 1.20E-11). KFD: 10 mg/kg ketamine, 0.01 mg/kg fentanyl, and 0.1 mg/kg dexmedetomidine; PNF: 2 mg/kg PAM, 0.1 mg/kg nalmefene, and 0.4 mg/kg flumazenil. Significant differences were determined by Student’s *t*-test (two-*t*ailed), mean ± SD, n = 7, male. ***P < 0.001 vs. KFD.

Given the role of PAMs in the control of NMDA receptor activity^9–11^, we investigated whether GNE-5729 (Fig. 1C), a potent PAM of GluN2A-containing NMDA receptors, could reverse the ketamine-induced anesthesia. The rats were treated successively with ketamine (50 mg/kg) and the PAM (1–4 mg/kg), and the duration of LORR was recorded. We found that treatment with ketamine rapidly induced LORR in the rats (3.20 ± 0.30 min) (Supplementary Table S1), and the PAM dose-dependently decreased the duration of LORR induced by ketamine (Fig. 2A). Because no analgesic effect was observed at 50 mg/kg ketamine, pain indexes were not assessed in either the ketamine group or the ketamine + PAM group. These results suggested that the PAM could reverse the anesthesia induced by ketamine.

### PNF significantly reversed the KFD-induced anesthetic and analgesic effects

**Figure 3:**
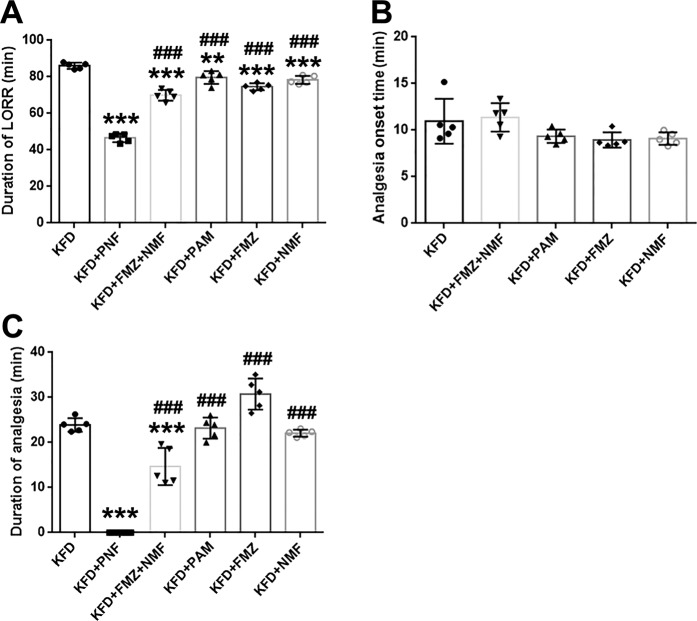
The effects of different sets of antagonists on the functions of KFD. After rats were anesthetized by KFD (10 mg/kg ketamine, 0.01 mg/kg fentanyl, and 0.1 mg/kg dexmedetomidine), antagonists or saline were administered once the righting reflex was lost, and the durations of LORR (**A**) and analgesia (**B**,**C**) were recorded. PNF: 2 mg/kg of PAM, 0.1 mg/kg nalmefene, and 0.4 mg/kg of flumazenil; FMZ + NMF: 0.4 mg/kg of flumazenil, and 0.1 mg/kg nalmefene; PAM: 2 mg/kg; FMZ: 0.4 mg/kg of flumazenil; NMF: 0.1 mg/kg nalmefene. Significant differences were determined by one-way analysis of variance, mean ± SD, n = 5, male. **P < 0.01, ***P < 0.001 vs. KFD; ^###^*P* < 0.001 vs. KFD + PNF.

To determine the effect of the PAM, nalmefene, and flumazenil combination (PNF) on the anesthesia and analgesia induced by KFD, we determined the duration of LORR and analgesia in the rats. The results shown in Fig. 2B revealed that PNF (48.15 ± 3.08 min) significantly reduced the KFD-induced duration of LORR (87.41 ± 3.01 min). Notably, we found that PNF even completely reversed the analgesic effect of KFD (Fig. 2C). The reversal effect of the PNF combination on the KFD-induced anesthesia was much stronger than that of the single drug or the flumazenil + nalmefene combination (Fig. 3A). Furthermore, the duration of LORR was similar between the three dose groups of nalmefene and flumazenil combinations (Supplementary Fig. S1A) and longer than that of the PNF group, suggesting that increasing the doses of nalmefene and flumazenil could not increase the efficacy of the reversal of the KFD-induced anesthesia without the PAM. The results indicated the critical role of the PAM in reversing the effect of KFD-induced anesthesia.

Moreover, the analgesia onset time was similar in all groups except that with the PNF combination (Fig. 3B), which completely inhibited the KFD-induced analgesia (Fig. 3C). As shown in Fig. 3C, administration of the PAM, nalmefene or flumazenil alone could not reduce the duration of the KFD-induced analgesia. The results revealed that these three drugs did not affect baseline analgesia when administered alone at the dose we used. These results demonstrated that PNF could effectively reverse the KFD-induced anesthetic and analgesic effects and offered better antagonistic effects than the three drugs alone or the combination of flumazenil and nalmefene. It seems that the synergistic effect of the PNF combination may be the main factor reversing the actions of KFD.

In addition, we further investigated the effects of PNF administered at a clinically relevant time. Rats injected with KFD were then treated with antagonists 30 min after LORR, and the duration of LORR and analgesia were recorded (Supplementary Fig. S1B). As shown in Supplementary Fig. S1C, compared with that of the (KFD)’ group (83.82 ± 2.57 min), the duration of LORR was shorter in the (KFD + PNF)’ group (52.03 ± 3.55 min), indicating that PNF could effectively reduce the LORR time when administered 30 min after LORR onset. Moreover, there was no difference between analgesia onset time in the (KFD)’ and (KFD + PNF)’ groups (Supplementary Fig. S1D) since KFD induced analgesia before PNF injection, as expected. Moreover, the duration of analgesia was similar between the two groups (Supplementary Fig. S1E) because the time to recovery from the KFD-induced analgesia was approximately 30 min. That is, when injected with PNF, the analgesic effect of KFD was almost completely eliminated.

### PNF combined with KFD had no obvious adverse effects on the cardiovascular and respiratory systems

In a previous study, we found that KFD did not cause side effects in the cardiovascular and respiratory systems of rats^7^. However, it is not clear whether PNF treatment will affect these systems. Here, we further detected the effects of successive administration of KFD and PNF on the cardiovascular and respiratory systems of rats. SpO_2_ and the heart and respiratory rates were measured during the anesthesia and a 30 min wake-up period. During anesthesia, the heart rate of the KFD + PNF group was lower than that of the control (Table 1). The duration of the low heart rate was consistent with the duration of LORR in the KFD combined with PNF group (Fig. 2B). During the wake-up phase, the heart rate gradually rose and returned to normal levels in the rats treated with KFD and PNF. Furthermore, SpO_2_ and the respiratory rate were not significantly different between the control and KFD + PNF groups during anesthesia and the wake-up period. These results indicated that the antagonistic effects of PNF on KFD did not trigger adverse cardiovascular and respiratory reactions.

**Table 1:** Physiological parameters during anesthesia and the wake-up period.

Physiological Parameters	Time (min)	CON	Under anesthesia	Wake-up	
			KFD +PNF	Time (min)	KFD +PNF
HR (brpm)	5	453.00 ± 0.82	311.67 ± 5.44***	2	383.00 ± 6.48
RR (bpm)		53.00 ± 8.98	53.00 ± 10.23		52.33 ± 5.68
SpO_2_ (%)		90.50 ± 1.02	90.63 ± 0.95		90.00 ± 0.98
HR (brpm)	10	445.67 ± 7.32	307.33 ± 0.94***	5	382.67 ± 5.79
RR (bpm)		52.00 ± 0.82	49.00 ± 4.97		45.00 ± 2.87
SpO_2_ (%)		90.17 ± 0.25	90.43 ± 1.02		89.27 ± 0.45
HR (brpm)	15	450.33 ± 5.44	312.00 ± 3.74***	10	388.00 ± 8.64
RR (bpm)		48.67 ± 4.03	56.67 ± 5.91		44.67 ± 3.47
SpO_2_ (%)		91.23 ± 1.05	84.87 ± 6.56		90.47 ± 0.29
HR (brpm)	25	448.33 ± 5.44	326.00 ± 12.96***	15	406.33 ± 11.90
RR (bpm)		52.67 ± 8.99	55.67 ± 4.50		56.33 ± 3.54
SpO_2_ (%)		90.87 ± 1.17	86.97 ± 5.07		91.00 ± 1.10
HR (brpm)	35	444.67 ± 2.05	324.00 ± 12.57***	20	411.67 ± 8.34
RR (bpm)		48.00 ± 4.97	56.00 ± 6.38		48.00 ± 6.13
SpO_2_ (%)		92.40 ± 0.22	91.17 ± 1.11		91.10 ± 0.28
HR (brpm)	45	449.67 ± 7.59	336.00 ± 23.34**	30	417.33 ± 6.65
RR (bpm)		49.00 ± 4.97	52.33 ± 9.84		48.67 ± 3.15
SpO_2_ (%)		90.73 ± 0.76	90.33 ± 0.33		90.67 ± 0.76

KFD: 10 mg/kg ketamine, 0.01 mg/kg fentanyl, and 0.1 mg/kg dexmedetomidine; PNF: 2 mg/kg PAM, 0.1 mg/kg nalmefene, and 0.4 mg/kg flumazenil; CON: saline injection. SpO_2_ and heart and respiratory rates were measured under anesthesia and a during a 30 min wake-up period. Significant differences were determined by one-way analysis of variance, mean ± SD, n = 6, male. ***P < 0.001 vs. CON. HR: heart rate; RR: respiratory rate.

### Hepatic and renal function examination

**Figure 4:**
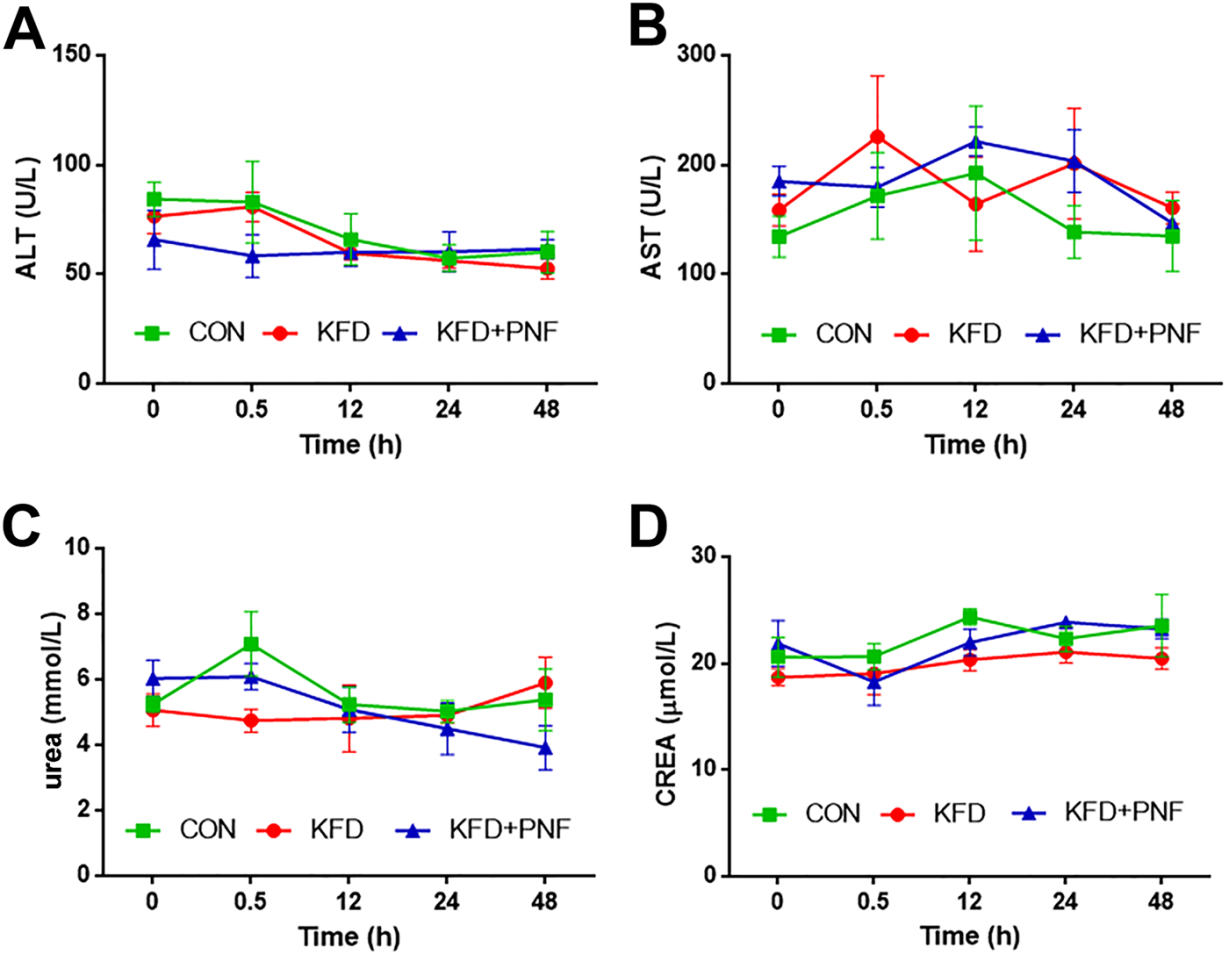
Detection of serum marker levels. Rats were administered KFD (10 mg/kg ketamine, 0.01 mg/kg fentanyl, and 0.1 mg/kg dexmedetomidine) and then PNF (2 mg/kg PAM, 0.1 mg/kg nalmefene, and 0.4 mg/kg flumazenil) 6 min later. Before KFD administration (0 h) and at 0.5, 12, 24 and 48 h after injection with KFD, the levels of serum ALT (**A**), AST (**B**), urea (**C**) and CREA (**D**) were assessed. Significant differences were determined by two-way analysis of variance, mean ± SD, male. CON: saline control group, n = 4; KFD, n = 4; KFD + PNF, n = 3. ALT: alanine aminotransferase; AST: aspartate transaminase; CREA: creatinine.

We then determined the effects of the KFD and PNF combination on the liver and kidney, which are important metabolic and excretory organs, respectively. As shown in Fig. 4, neither the KFD nor KFD + PNF-treated rats had notable changes in the serum ALT, AST, urea and CREA levels compared with those at the 0 h time point (before KFD administration). Moreover, there were no obvious differences in the serum marker levels between the control and KFD or KFD + PNF groups. Although the urea levels declined in the KFD + PNF group, this change was not significant. The results revealed that PNF combined with KFD had no evident impact on hepatic and renal function.

### PNF substantially decreased the duration of the KFD-induced loss of motor ability

**Figure 5:**
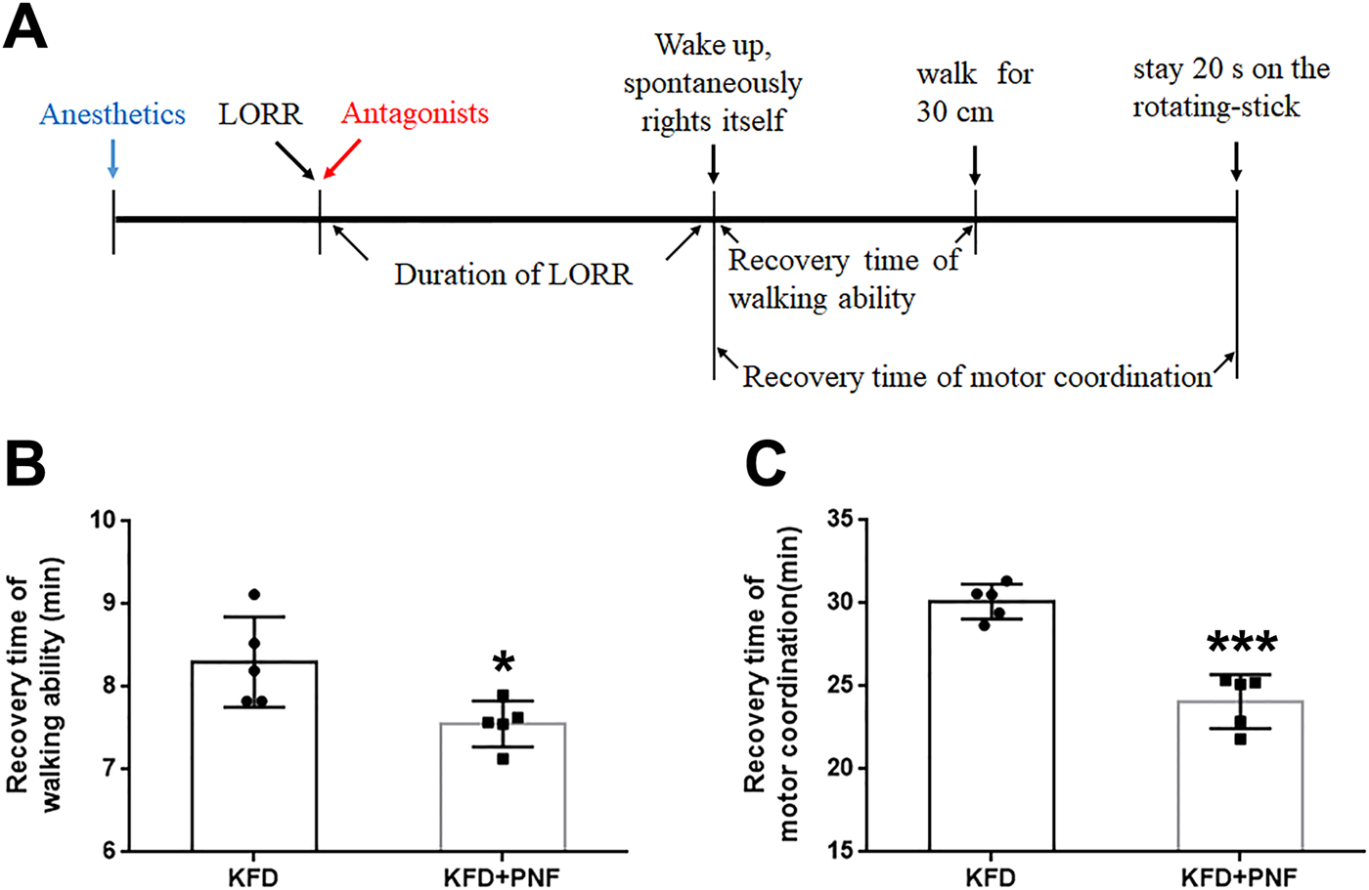
PNF decreased the duration of the KFD-induced loss of motor ability. (**A**) Schematic diagram of the rotarod test design. The time to recover the walking ability (**B**), (*P* = 0.0255) and motor coordination (**C**), (*P* = 1.18E-04) was determined. Significant differences were determined by Student’s *t*-test (two-tailed), mean ± SD, n = 5, male. *P < 0.05, ***P < 0.001 vs. KFD.

Motor coordination, strength and balance after anesthetic and antagonist administration were assessed by the rotarod test (Fig. 5A). We found that the time to regain walking ability in the KFD + PNF group was shorter than that in the KFD group (Fig. 5B). In addition, the KFD + PNF-treated rats presented obviously higher motor performance in the rotarod test than the KFD-treated rats (Fig. 5C). These results suggested that PNF could improve the recovery of the motor ability.

### PNF combined with KFD had no influence on cognitive function

**Figure 6:**
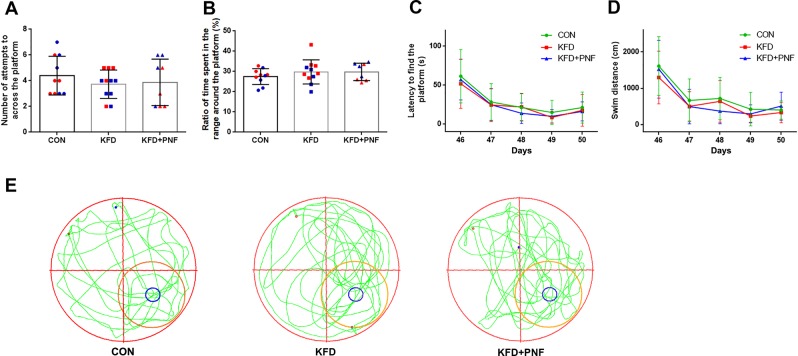
KFD and PNF had no effect on the cognitive function of rats. Rats at 5 weeks of age underwent the first MWM test (Days 1–5), and then, they were administered the drugs (Day 16). For KFD + PNF treatment, the rats received PNF administration once they lost the righting reflex after KFD injection. A month later, the second round of the MWM test (Days 46–50) was performed. The number of attempts to across the platform (**A**) and the ratio of time spent in the range around the platform (**B**) of the second round MWM were similar between the control and KFD or KFD + PNF groups. (**A**,**B**) The blue individual data represent female rats, and the red data represent male rats. There were no differences in the latency to find the platform (**C**) or swim distance (**D**) in the three groups. (**E**) Representative traces of the movement of the rats in the spatial probe test (Day 50). The blue circle represents the removed platform, and the orange circle represents the range around the platform. Significant differences were determined by two-way analysis of variance, mean ± SD. CON: saline control group, n = 10; KFD, n = 11; KFD + PNF, n = 8.

Growing evidence suggests that prolonged exposure to general anesthetics induces widespread neurodegeneration and long-term cognitive deficits^25,32,33^. Therefore, we investigated whether successive administration of KFD and PNF could result in learning and memory abnormalities. We performed two rounds of MWM tests on the rats to evaluate their learning and memory skills before and after the drug treatment. As shown in Supplementary Fig. S2A–E, there was no difference in the three groups in the first round of the MWM test (Days 1–5), which indicated that the rats from different experimental groups had similar cognitive functions before drug administration. The rats were then allowed to recover for 10 days to minimize the impact of the behavioral tests. Then, they were administered saline or drugs (Day 16) and underwent the second round of the Morris water maze (Days 46–50) 1 month after drug administration to evaluate the long-term impacts of the cocktails. We found that the KFD or KFD + PNF group performed similarly compared with the control group in the MWM test (Fig. 6). There was no significant difference in the number of attempts to across the platform or the ratio of time spent in the range around the platform among the three groups (Fig. 6A,B). The latency to find the platform and the swim distance of the KFD and KFD + PNF groups on the testing day were similar to those of the control group (Fig. 6C,D). Representative traces of the movement of the rats in the spatial probe test at the end of the training period are also shown, with the platform circled in blue and the range around the platform marked in orange (Fig. 6E). In addition, we found that the latency to find the platform and the swim distance of the KFD and KFD + PNF groups on Day 46 were similar to those of the control group (Fig. 6C,D), and these values were both significantly shorter than those when they were first trained on day 1 (Supplementary Fig. S2F,G), indicating that the drug cocktail has no effects on memory retention. These results indicated that KFD or PNF did not impact the cognitive function of the rats at the tested age.

Because both male and female rats were used in the MWM tests, to verify whether sex could affect the results of MWM, we further tested the response to anesthetics and evaluated the potential impacts of anesthetics on cognitive function with the Morris water maze for both sexes. We found that rats from both sexes were similarly anesthetized by the cocktail (Supplementary Fig. S3A,B), and the cognitive function of both genders was not altered by either cocktail (Supplementary Fig. S3C–F). These results suggest that animals from both genders respond similarly in our study.

## Discussion

In a previous study, although we showed that KFD was a safe and effective anesthetic formulation, the duration of the KFD-induced LORR was much longer than that of ketamine alone^7^, which indicated that KFD may not be suitable for short-term surgery. Therefore, reversing anesthesia with antagonists may be a method with a high anesthetic quality and a short duration of anesthesia. However, to date, there are no drugs that can effectively antagonize the anesthesia induced by ketamine clinically. Ketamine is a commonly used pediatric anesthetic that mainly antagonizes NMDA receptors^34,35^. PAMs are novel pharmacological tools to enhance NMDA receptor activity that act at distinct sites compared with NMDA receptor agonists and do not directly activate the receptors^9,36,37^. Whether PAMs could affect KET-induced anesthesia is unclear. In the present study, GNE-5729, a recently discovered GluN2A subunit-selective PAM^8^, was shown to dose-dependently reduce ketamine-induced LORR.

Fentanyl, a potent opioid analgesic, induces analgesia and anesthesia primarily via activation of the μ-receptor^38^. The highly selective α2-adrenoceptor agonist dexmedetomidine is an effective adjuvant medicine in clinical anesthesia due to its analgesic, sedative, and anxiolytic activity^39^. Naloxone, an opioid receptor antagonist, is currently the only approved treatment for opioid overdose by the US Food and Drug Administration (FDA). However, several clinical case studies suggest that fentanyl overdose may require higher doses of naloxone to reverse the effects of the overdose^40^. Recently, NIH leadership advocated the development of more effective antagonists^41^. Many studies have demonstrated that the affinity for µ-opioid receptors of nalmefene is approximately 5 times higher than that of naloxone. Moreover, nalmefene has a similar half-life to that of fentanyl^42,43^. According to Dougherty *et al*., nalmefene could partially reverse analgesia in patients receiving epidural fentanyl in dilute bupivacaine for postoperative pain control^19^. Nalmefene may be a potent candidate to antagonize the effects of KFD. Although it has been reported that flumazenil could counteract the impairment of ketamine on recognition memory in rats^23^, the role of flumazenil in ketamine-induced anesthesia remains unclear. In this report, we found that the PAM combined with nalmefene and flumazenil significantly decreased the duration of sedation and analgesia induced by KFD. Furthermore, the addition of the PAM to nalmefene and flumazenil had a stronger antagonistic effect on KFD in rats than the single drug or combination of nalmefene and flumazenil.

The anesthetics used in our formula have been reported to be associated with cardiovascular and respiratory systems^2,38,44^. To evaluate the cardiovascular and respiratory system effects of the combination of KFD and PNF, we further determined the SpO_2_ and heart and respiratory rates of the rats. Successive administration of KFD and PNF led to a lower heart rate during anesthesia, which gradually returned to normal levels after awakening. We observed that the low heart rate of the KFD + PNF group was comparable to that of KFD in our previous study^7^. Moreover, there were no significant changes in the SpO_2_ or respiratory rate of the KFD + PNF group during the detection period compared with those of the control. These results suggested that the addition of PNF was safe for the cardiovascular and respiratory systems. Considering the novel roles of the liver and kidney in drug metabolism and excretion, respectively, we validated the effects of the KFD and PNF combination on the two organs. The results demonstrated that PNF combined with KFD did not evidently change serum marker levels, indicating no effect on hepatic and renal function.

The impact of anesthetic exposure on neurodevelopment has been a focus of attention. Multiple studies have suggested that prolonged exposure to general anesthetics results in accelerated neurodegeneration and neurocognitive deficits in neonatal rats^25,26^. However, the GluN2A subunit starts to be expressed significantly after the first two postnatal weeks^27,28^. Because of this, the neonatal brains lack enough targets for the PAM, and our antagonist cocktail should not work in these animals. Indeed, our results validated this prediction, and the PAM could not reverse the ketamine-induced anesthesia in neonatal rats (Supplementary Fig. S1F). In addition, the safety of combined anesthetics in young adults remains unclear. Additionally, it is not clear whether successive administration of KFD and PNF could result in learning and memory abnormalities. Therefore, we examined the safety of both cocktails and performed experiments in 5-week-old rats for verification. We found that administration of PNF decreased the recovery time of the motor ability in the KFD group. Furthermore, there were no differences in learning and memory among the control, KFD, and KFD + PNF groups. Moreover, both sexes responded to anesthetics similarly, and the cognitive function of both genders was not altered by either cocktail. Collectively, KFD or PNF did not impact brain function.

In this study, we propose a new antagonistic formulation consisting of a PAM, nalmefene, and flumazenil in rats. The aim of this study was to shorten the duration of anesthesia and enhance recovery. Interestingly, we also observed reversal of analgesia. This finding suggests a common pathway/mechanism underlying anesthesia and analgesia from our cocktail. It would be useful to develop drugs that only impact anesthesia without resulting in resensitization to pain after surgery. In addition, we found that treatment with PNF 30 min after the KFD-induced LORR could only decrease the duration of LORR and had no effects on the analgesia induced by KFD because the time to recovery from the KFD-induced analgesia was approximately 30 min. These results suggested that the analgesic effect of KFD might have already disappeared within 30 min before PNF administration. Although the combination of drugs may be beneficial to minimize the negative effects of each drug and to enhance the positive effects of the drugs, its effects are some time difficult to explain because of other factors. For example, the pharmacokinetics or/and pharmacodynamics of the drugs in combinations may change due to drug-drug interactions, which could alter the drug effects. The data in the current study do not support this possibility but could not completely rule it out. Thus, more work is needed to further clarify the effects of drug cocktails. The anesthetics we chose are all routinely used in the clinic in our department’s medical practice, and their doses in the cocktail were significantly lower than their doses when used individually. Therefore, we believe they have the potential to be used in the clinic in the future, which might reduce side effects caused by high doses of individual drugs. However, these factors need to be assessed in preclinical or clinical studies in the future.

In conclusion, we have demonstrated that a PAM dose-dependently reduced the duration of the ketamine-induced LORR, and the PAM combined with nalmefene and flumazenil effectively reversed the anesthetic and analgesic actions induced by ketamine-fentanyl-dexmedetomidine. Additionally, administration of PNF decreased the time to recover the motor ability after KFD. Successive administration of PNF and KFD had no effect on the hepatic, renal, and cognitive functions of the rats. These data suggested that PNF was an appropriate treatment for the antagonism of KFD.

## Supplementary information

Supplementary Information.

## Data Availability

All data generated or analyzed during this study are included in this published article and its Supplementary Information Files.
