# Supplementary material for: GluN2A-selective positive allosteric modulator-nalmefene-flumazenil reverses ketamine-fentanyl-dexmedetomidine-induced anesthesia and analgesia in rats
Source: Sci Rep. 2020 Mar 24;10:5265. doi: 10.1038/s41598-020-62192-8 (PMC7093419; doi:10.1038/s41598-020-62192-8)
Supplement: Supplementary file 1 — Supplementary Information. [file 41598_2020_62192_MOESM1_ESM.pdf]

GluN2A-selective positive allosteric modulator-nalmefene-flumazenil reverses ketamine-fentanyl-dexmedetomidine-induced anesthesia and analgesia in rats

Chunzhu Li [a, 1], Jia Yan [a, 1], Dewei Tang [d, e], Jidong Zhu [b, c], Chen Huang [f], Yu Sun [a], Rong Hu [a], Hao Wang [a], Chaoying Fu [b, c], Yelin Chen [b, c, **], Hong Jiang [a, *]

[a] *Department of Anesthesiology, Shanghai Ninth People's Hospital, Shanghai Jiao Tong University School of Medicine, Center for Specialty Strategy Research of Shanghai Jiao Tong University China Hospital Development Institute, Shanghai 200011, China*

[b] *Interdisciplinary Research Center on Biology and Chemistry, Shanghai Institute of Organic Chemistry, Chinese Academy of Sciences, Shanghai 201210, China*

[c] *University of Chinese Academy of Sciences, Beijing 100049, China*

[d] *Center for Molecular Imaging, Shanghai University of Medicine & Health Sciences, Shanghai, China*

[e] *Department of Nuclear Medicine, Renji Hospital, School of Medicine, Shanghai Jiao Tong University, 160 Pujian Road, Pudong New District, Shanghai, 200127, China*

[f] *College of Medical Imaging and Shanghai Key Laboratory of Molecular Imaging, Shanghai University of Medicine and Health Sciences, Shanghai, 201318, China*

[1] These authors contributed equally to this work.

* Corresponding author. Department of Anesthesiology, Shanghai Ninth People's Hospital, Shanghai Jiao Tong University School of Medicine, Center for Specialty Strategy Research of Shanghai Jiao Tong University China Hospital Development Institute, 639 Zhizaoju Road, Shanghai 200011, China, Tel.: +86-21-23271699, Fax: +86-21-23271699.

** Corresponding author. Interdisciplinary Research Center on Biology and Chemistry, Shanghai Institute of Organic Chemistry, Chinese Academy of Sciences, 26 Qiuyue Road, Shanghai 201210, China, Tel.: +86-21-68582363.

E-mail addresses: jianghongjiuyuan@163.com (H. Jiang), chenyelin@sioc.ac.cn (Y. Chen).

Table S1 Time of anesthesia onset after injected with anesthetics

Group	Induction time (min)
KET	3.20±0.30
KFD	5.56±0.38

KET: 50 mg/kg ketamine, n=5. KFD: 10 mg/kg ketamine, 0.01 mg/kg fentanyl, and 0.1 mg/kg dexmedetomidine, n=7. The data were presented as mean ± SD.

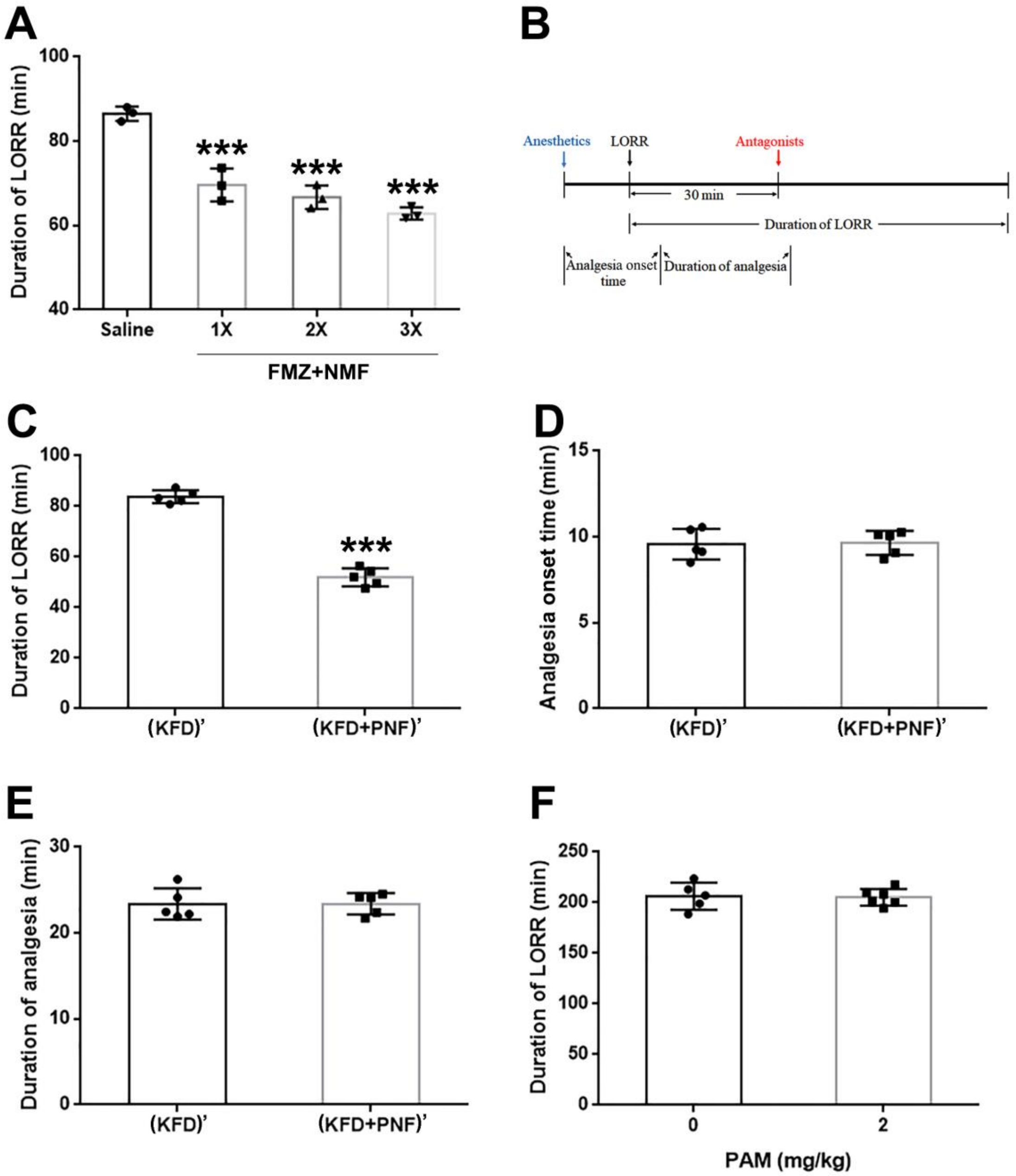

Fig. S1 (A) Anesthetic effects in 5-6W SD rats after injected with different dose combinations of flumazenil and nalmefene. After rats were anesthetized by KFD (10 mg/kg ketamine, 0.01 mg/kg fentanyl, and 0.1 mg/kg dexmedetomidine), antagonists or saline were administrated once the righting reflex lost, and the duration of LORR were recorded. FMZ+NMF: 1X: 0.1 mg/kg nalmefene and 0.4 mg/kg flumazenil; 2X: 0.2 mg/kg nalmefene and 0.8 mg/kg flumazenil; 3X: 0.3 mg/kg nalmefene and 1.2 mg/kg flumazenil. Significant differences were determined by one-way analysis of

variance, n=3, mean ± SD. ***, $P<0.001$ vs. Saline (KFD) group. (B) Schematic diagram of the evaluation of anesthesia. Rats injected with KFD, then treated with antagonists at 30 min after LORR, and the duration of LORR (C) and analgesia were recorded (D-E). (F) PAM could not reverse the ketamine-induced anesthesia in neonatal rats. After rats (P6) were anesthetized by ketamine (50 mg/kg), PAM (2 mg/kg) or saline were administrated once the righting reflex lost.

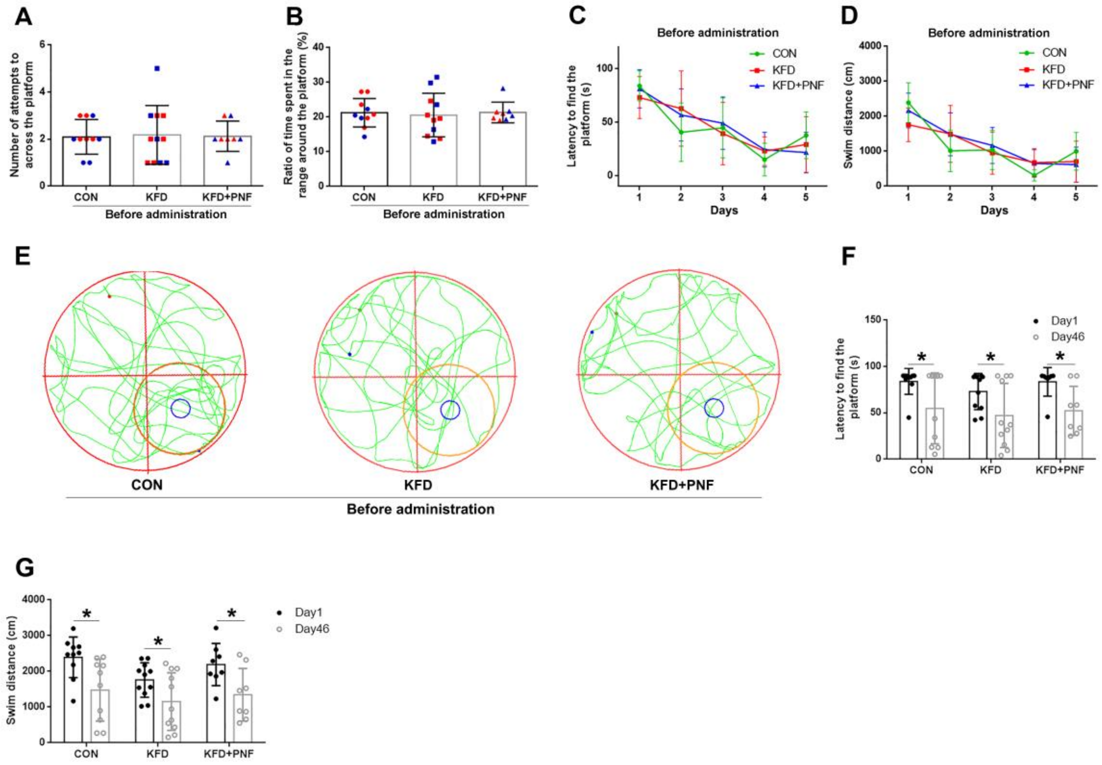

Fig. S2 The learning and memory abilities of all animals before administration were at the same level. The blue individual data represent female rats, and the red represents male rats (A-B). There were no differences in the number of attempts to across the platform (A), ratio of time spent in the range around the platform (B), latency to find the platform (C), and swim distance (D) of the first round MWM in the three groups. (E) Representative traces of the movement of rats in the spatial probe test (Day 5). The blue circle represents the removed platform, and the orange represents the range around the platform. Significant differences were determined by two-way analysis of variance, mean ± SD. The latency to find the platform (F) and swim distance (G) of KFD and KFD + PNF groups on Day 46 were both significantly shorter than when they were first trained in day 1. Significant differences were determined by Student's *t*-test (two-tailed), mean ± SD. CON: saline control group, n=10; KFD, n=11; KFD+PNF, n=8. *$P < 0.05$.

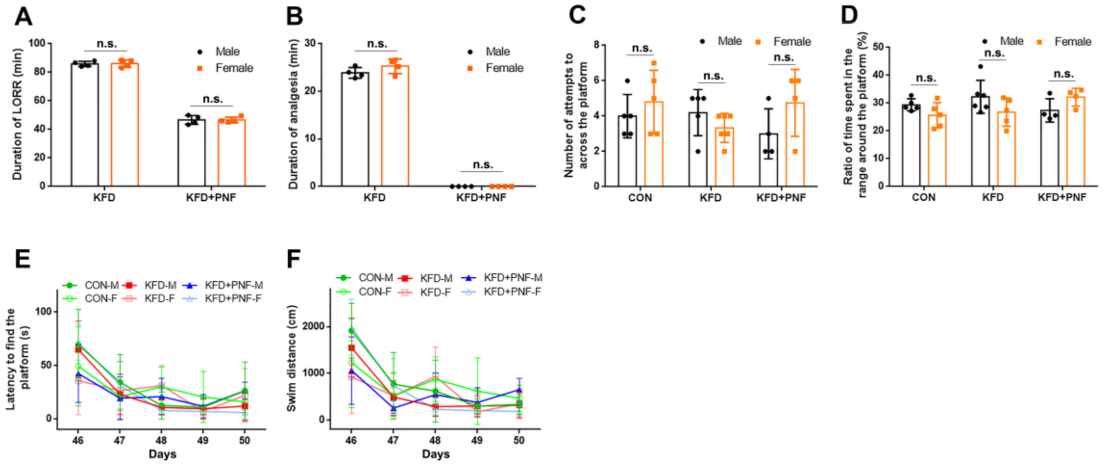

Fig. S3 (A-B) After rats were anesthetized by KFD (10 mg/kg ketamine, 0.01 mg/kg fentanyl, and 0.1 mg/kg dexmedetomidine), antagonists or saline were administrated once the righting reflex lost. Both sexes responded to anesthetics the same way. (C-F) There were no differences in the learning and memory abilities of male and female rats in each group.
